# The roles of health culture and physical environment in workplace health promotion: a two-year prospective intervention study in China

**DOI:** 10.1186/s12889-018-5361-5

**Published:** 2018-04-05

**Authors:** Yingnan Jia, Hua Fu, Junling Gao, Junming Dai, Pinpin Zheng

**Affiliations:** 1 School of Public Health, Key Lab of Public Health Safety of the Ministry of Education, Fudan University, Shanghai, 200032 China; 2 Health Communication Institute, Fudan University, Shanghai, 200032 China

**Keywords:** Workplace health promotion, Health culture, Physical environment, Intervention implementation, Mediating effect

## Abstract

**Background:**

To understand the potential influencing factors on the effectiveness of workplace health promotion interventions and examine whether workplace health culture and physical environment can mediate the relationship between workplace health promotion and intervention effectiveness.

**Methods:**

A total of 719 participants from 10 Chinese government agencies were recruited for a prospective self-controlled trial. Questionnaires, qualitative interviews, and direct observation were used for the baseline evaluation, process evaluation, and effectiveness evaluation. Based on the results of the need assessment and risk assessment at each workplace, a two-year comprehensive health intervention was conducted by each workplace. Health outcomes including self-rated health (SRH) and mental health were measured at baseline and 24 months. Health culture was measured at 24 months. Physical environment and intervention implementation were measured at 12 months and 24 months.

**Results:**

Compared with the baseline, the means of SRH and mental health increased significantly by 0.302 and 2.698, respectively. The SRH scores were different before and after intervention; furthermore, the differences varied by workplace. Health culture mediated the relationship between intervention implementation and intervention effectiveness, including SRH and mental health improvement, but physical environment did not. Physical environment quality was significantly negatively correlated with SRH improvement and mental health improvement. Under the relatively high-quality interventions with scores higher than 4.047 or 4.151 (out of 5), better health culture may led to greater SRH and mental health improvements.

**Conclusions:**

Health culture may mediate the relationship between intervention implementation and intervention effectiveness, whereas physical environment does not seem to mediate this relationship. Under relatively high-quality interventions, a better health culture may lead to more positive improvements in SRH and mental health. Future studies will need to examine the physical environment as a moderating effect rather than mediating effect.

**Trial registration:**

This study was retrospectively registered in Chinese Clinical Trial Registry. Trial registration number: ChiCTR-OOC-16010059. Date of registration: Dec 1, 2016.

**Electronic supplementary material:**

The online version of this article (10.1186/s12889-018-5361-5) contains supplementary material, which is available to authorized users.

## Background

The employed population is society’s most valuable resource due to its vitality, creativity, and productivity. The health of the employed population directly affects the survival and development of enterprises and is associated with national economic development, progress, and social stability. A number of unhealthy behaviors can impair the functioning and productivity of people between 18 and 65 years of age. Previous studies showed that the loss of productivity in the employed population due to personal or family health problems was very large. In the United States, a survey of 28,000 people showed that the annual loss of productivity due to employee health problems was nearly 226 billion US dollars [1]. The annual economic loss caused by depression alone can reach 44 billion US dollars [2]. Hence, protecting the health of the employed population has become a major problem for many countries. The World Health Organization (WHO) suggests that workplaces should play a key role in health promotion [3]. The workplace is an ideal setting for health promotion for a number of reasons: adults spend a considerable portion of their time at work; the working population is relatively stable, suitable for long-term health interventions and follow-up; workplaces can provide space and infrastructure for participants; the physical and psychological environment of the workplace is an important influencing factor on employees’ health; and working people in good health can improve their work efficiency [4, 5]. The United States Centers for Disease Control and Prevention (CDC) also promotes health promotion programs in the workplace because effective health promotion programs benefit employers, employees, employees’ families, and communities [6].

Several studies have also confirmed the value of workplace health promotion programs, finding that such programs improve employees’ health knowledge, attitudes, and behaviors, improve the health of employees, and reduce potential health problems, health care costs, and absenteeism [4, 7, 8].

A Healthy Workplace Model was proposed by WHO in 2010, which identified employee needs including physical work environment, psychosocial work environment, personal health resources and ways of participating in the community [9]. However, the potential influencing factors and underlying mechanisms of health promotion effectiveness remain unclear. Without understanding these factors, it is difficult to improve the effectiveness of workplace health promotion efforts. Thus, researchers have begun to examine the impact of a number of potential influencing factors, such as workplace culture, physical environment, and intervention implementation. Golaszewski et al. [10] pointed out the importance of creating the cultural and supportive environment in workplace health promotion. A qualitative study by Waterworth et al. [11] found that improving the culture and physical environment of a workplace had a significant impact on health promotion. Hall et al. [12] revealed that the participation rate of health promotion programs might be positively associated with the workplace culture. Several studies confirmed that the frequency and characteristics of intervention implementation may be related to health promotion effectiveness [13]. However, most of the existing studies were qualitative studies and lacked the support of convincing quantitative data. As noted by Wierenga et al. [14], most workplace health promotion programs lacked a systematic, high-quality process assessment, making it difficult to assess the relationship between intervention implementation and outcomes.

To improve the effectiveness of workplace health promotion, it is necessary to explore how potential influencing factors affect health promotion effectiveness. According to the Healthy Workplace Model, workplace health culture, physical environment, and intervention implementation may be associated with intervention effectiveness [9]. In this study, we investigated the influencing factors on intervention effectiveness and examined whether workplace culture and physical environment could mediate the relationship between workplace intervention (implementation of overall intervention and specialized programs) and health promotion effectiveness (SRH improvement and mental health improvement) using a prospective self-controlled design with 10 government agencies. We used questionnaires, qualitative interviews, and direct observation for the baseline, process, and effectiveness evaluations. We also developed new scales for quantitatively evaluating workplace health culture, physical environment, and intervention implementation through a systematic process assessment.

## Methods

### Study design

Written informed consent statement forms were obtained from participants. The right to withdraw and autonomy of responses were also explained. This study received approval from the ethics committee of the Fudan University School of Public Health, China. A prospective self-controlled trial was carried out between 2012 and 2014 in Shanghai. Primary outcomes, including SRH and mental health, were measured at baseline and after 24 months. Health culture was measured at 24 months. Physical environment and intervention implementation were measured at 12 months and 24 months. Questionnaires, qualitative interviews, and direct observation were used for the baseline evaluation, process evaluation, and effectiveness evaluation. The study began on June 15, 2012, and follow-up continued until September 30, 2014. Please refer to Additional file 1 for details of the study protocol.

### Study population

Participants were recruited from 10 government agencies in Shanghai, China. The inclusion criteria for participants were (1) having signed an employment contract and (2) having signed a written informed consent form. A total of 1007 participants were recruited for the study, of whom 719 (71.4%) completed the questionnaire at the baseline and final follow-up.

### Intervention

#### Theoretical basis

The intervention was developed based on the WHO Healthy Workplace Model [9]. We identified four areas—physical work environment, psychosocial work environment, personal health resources, and enterprise community involvement—in which actions towards a healthy workplace could be taken, and seven steps towards achieving effective health promotion. Step 1 is to mobilize employers and employees to invest in change. Step 2 is to help them assemble a “healthy workplace team” and resources to work on implementing a particular change in the workplace. Step 3 is to develop an assessment to collect the baseline data on employee demographics, health status, lifestyle, and psychosocial work environment (for example, job demand and job control). Step 4 is to determine the priorities that are most essential to health. Step 5 is to devise a health plan. Step 6 is to conduct the intervention. Step 7 is to evaluate the implementation process as well as the outcomes. Step 8 is to improve the program based on the evaluation results.

#### Procedure

Considering the characteristics of civil servants, the development of the detailed program plan was based on four key steps:

1. At the start of the program, we recruited the top management of each government agency that committed to the program. The agencies agreed to invest essential human and financial resources into health promotion.
2. In the baseline survey, need assessment was conducted in each workplace to collect the baseline data and explore the employees’ needs, including the content, pattern, time, and frequency of intervention. Each workplace received a report on the baseline investigation, which included the employees’ demographic characteristics, health statuses, lifestyles, work-related characteristics, and health promotion demand. Some priority problems were determined based on prevalence, correlation with other risk factors, impact potential, and consistency between management’s ideas and the views of employees.
3. Based on investigation of the literature, a template for an intervention program was designed by the researchers. The program consisted of four main parts, which focused on tobacco control, physical activity, nutrition and diet, and work stress. Each workplace then designed an intervention plan based on the actual conditions and baseline data of that workplace. Each plan was improved by the researchers.
4. Each workplace assembled a “healthy workplace team”, which had several members and received financial support dedicated to health promotion. Focusing on the key problems, each workplace made targeted efforts to promote employees’ health. Some common interventions were as follows: For tobacco control, each workplace enacted measures such as banning smoking indoors, banning smoking in the office, having managers lead by example by not smoking themselves, and posting no-smoking signs. For physical activity, space was expanded, more exercise facilities were provided, interest groups were organized and given financial support, and employees were encouraged to walk rather than take the elevator by posters hung at decision points. For nutrition and diet, salt and oil dosage were recorded by the employer every working day; healthy eating habits were introduced by means such as lectures, leaflets, posters, and videos; and food intake recommendations were offered using color codes (red for limited intake; yellow for appropriate intake; green for recommended intake). For stress management, social activities were organized and lectures on mental health were held.

### Measures

#### Demographic variables

Self-reported demographic variables included gender, age, marital status, education, and length of employment. Age, education, and length of employment were each separated into four categories. The four age categories were < 30, 30–39, 40–49, and ≥ 50. The four education categories were junior high school, high school/technical secondary school, junior college, and bachelor/master’s/doctorate degree. The four length of employment categories were < 5 years, 5–14 years, 15–24 years, and ≥ 25 years.

#### Primary outcome measures

##### Self-rated health

SRH was one of the main health outcomes we examined. In both the baseline and final survey, respondents were asked to rate their own general health on a five-point scale ranging from perfect to poor. SRH was generally assessed by a single survey question inviting participants to provide a subjective assessment of their health. Respondents answered “would you say that in general your health is perfect, very good, good, fair, or poor?” on a 5-piont scale ranging from perfect to poor [15]. The difference between the baseline and final data was included in the analysis as a continuous dependent variable.

##### Mental health

The other main health outcome, mental health, was measured by the Chinese version of the WHO-Five Well-Being Index. The WHO-5 has been found to be reliable and valid in previous studies [16, 17]. Respondents were asked to rate their status over the past 14 days. For example, how often have you felt quiet and relaxed [16]? Each item was rated on a six-point scale ranging from 0 (never) to 5 (all the time). The difference between the baseline and final scores was included in the analysis as a continuous dependent variable.

#### Potential influencing factors on intervention effectiveness

##### Implementation of intervention

We developed the Chinese Workplace Health Scorecard based on the CDC Workplace Health Scorecard [18], with adjustments based on the Chinese context. We organized 10 personal qualitative interviews to collect data about the implementation of intervention from the health promotion staff of each workplace. During the interviews, The Scorecard was filled out by the health promotion staff of each workplace to evaluate overall health promotion and specialized health promotion. Overall health promotion was assessed by 12 questions that focused on need assessment and health promotion programs. Each item was rated on a scale from 1 to 5, with a higher score indicating better overall health promotion. Specialized health promotion was divided into five parts: tobacco control, diet, physical activity, weight control, and stress management. Each part included several items rated on a scale from 1 to 5, with a higher score indicating better specialized health promotion. The average of the overall and specialized health promotion scores was calculated to obtain the intervention implementation score and included in the analysis as a continuous variable.

##### Workplace health culture

Based on the Chinese context, we developed the Workplace Health Culture Scale. The scale included 20 items divided into five dimensions: individual health culture, adverse health behaviors of direct leadership, adverse health effects of direct leadership, beneficial health effects of direct leadership, and overall health culture [19]. More detailed information about the items can be found in another article [20]. Each item was rated on a scale from 1 to 5, with a higher score indicating better workplace health culture. Each of the five domains was measured by the average of the ratings of the items. The scores on the five dimensions were averaged to obtain the overall score, which was included in the analysis as a continuous variable. The effects of workplace health culture were derived from the intervention effectiveness evaluation.

##### Workplace physical environment

The workplace physical environment varied among workplaces, but did not vary among individuals in the same workplace. Therefore, the workplace physical environment was assessed by direct observation. There were four trained observers who were responsible for visiting each workplace and using the Direct Observation Scoring Table to record their observations. During the field visit, the health promotion staff of each workplace guided the observers and answered the necessary questions. The assessment content of the Direct Observation Scoring Table was divided into four parts: overall environment, physical activity environment, tobacco control environment, and nutritional/dietary environment. Each part consisted of 3 items rated on a scale from 1 to 5, for a maximum total of 60 points. The mean of the total scores among the observers was computed as the physical environment score of each workplace. The average value of the process evaluation and effectiveness evaluation was included in the analysis as a continuous variable. Please refer to Additional file 2 for details of Direct Observation Scoring Table.

### Quality control

#### Questionnaire investigation

The questionnaires were self-filled by the respondents. Trained investigators were responsible for on-site quality control, including answering questions and asking respondents to complete any missing items on the questionnaires.

#### Direct observation

Using the Direct Observation Scoring Table, each trained observer rated the health environment of the workplace independently through field observation.

### Analyses

Descriptive analyses, *t* tests, and multiple regressions were conducted for the quantitative data using Epidata 3.1, Excel 2010, and Statistical Package for Social Sciences 20.0. To evaluate the effectiveness of an intervention, first, a paired *t* test was used; then, repeated measurement and analysis of variance were performed. Multiple regression analysis was conducted to determine whether health culture and physical environment could mediate the relationship between intervention implementation and intervention effectiveness.

## Results

### Demographics of the participants

Of the original 1007 participants, 719 participants completed both the baseline and final survey, with a 26.3% participation loss at follow-up. The data of these 719 participants were used to evaluate the intervention effectiveness. Based on the baseline data, the demographics of the study sample are reported in Table 1. In the final sample, 52.3% of participants were male and 47.7% were female. Over 60% of participants were between 30 and 49 years of age. Nearly 90% were married, nearly half had 5 to 14 years of work experience, and 58.4% had a bachelor’s or higher degree. More information on the demographic characteristics of workers from each organization was provided in Additional file 3.

**Table 1:** Demographics of the participants

Variables		N (%)	Variables		N (%)
Gender	Male	376 (52.3)	Education	Junior high school	54 (7.5)
	Female	343 (47.7)		High school/technical secondary school	75 (10.4)
Marital status	Married	643 (89.4)		Junior college	170 (23.6)
	Unmarried/divorced/widowed	76 (10.6)		Bachelor or higher degree	420 (58.4)
Age, years	< 30	129 (17.9)	Length of employment, years	< 5	201 (28.0)
	30–39	222 (30.9)		5–14	346 (48.1)
	40–49	226 (31.4)		15–24	120 (16.7)
	≥50	142 (19.7)		≥25	52 (7.2)

The data were based on the baseline survey results from 2012

### Self-rated health and mental health before and after intervention

Table 2 showed that the mean of baseline SRH was 2.96 ± 0.83, while the mean of final SRH was 3.26 ± 0.97. The mean of SRH increased by 0.302 between the baseline and final survey. The mean of baseline mental health was 14.79 ± 5.02, while the mean of final mental health was 17.49 ± 4.92. The mean of mental health increased by 2.698 between the baseline and final survey. The differences were statistically significant. More information on the SRH and mental health of workers from each organization was provided in Additional file 3.

**Table 2:** Paired *t* test: Self-rated health and mental health before and after intervention

Variables		Mean	*t*	*P*
Self-rated health (*N* = 712)	Self-rated health (baseline)	2.96 ± 0.83		
	Self-rated health (final)	3.26 ± 0.97		
	Self-rated health (final) − self-rated health (baseline)	0.302 ± 1.045	7.712	< 0.001
Mental health (*N* = 696)	Mental health (baseline)	14.79 ± 5.02		
	Mental health (final)	17.49 ± 4.92		
	Mental health (final) − mental health (baseline)	2.698 ± 5.977	11.911	< 0.001

Considering the different intervention implementations among workplaces, repeated measures ANOVA was performed with the workplace as a block factor in the analysis. Table 3 shows that the SRH scores before and after intervention differed, and the degree of difference varied by workplace. The differences were statistically significant. Similar results were found for mental health.

**Table 3:** Repeated measures ANOVA: Self-rated health and mental health before and after intervention

Variables (N)		*SS* (III)	*df*	*MS*	*F*	*P*
Self-rated health (*N* = 712)	Self-rated health	30.407	1	30.407	58.898	< 0.001
	Self-rated health × workplace	25.616	9	2.846	5.513	< 0.001
	Error	362.423	702	0.516		
Mental health (*N* = 696)	Mental health	2369.790	1	2369.790	149.081	< 0.001
	Mental health × workplace	1507.693	9	167.521	10.539	< 0.001
	Error	10,904.626	686	15.896		

### Testing the mediating effects of health culture and physical environment on self-rated health and mental health improvement

We conducted eight regression logistic models to test the mediating effects of health culture and physical environment. The dependent variable for regression logistic models A, B, C, and D was self-rated health improvement, while the dependent variable for regression logistic models E, F, G, and H was mental health improvement. The independent variables of models A, C, E, and G included age, gender, education level, marital status, intervention implementation and one of the two potential mediating variables. The interaction was introduced into models B, D, F, and H. Table 4 shows that only health culture mediated the relationship between intervention and self-rated health and mental health improvement. Therefore, there are only model A, D, E, and H presented in Table 5 and Table 6. Please refer to Additional file 3 for more information about intervention implementation, workplace health culture, and workplace physical environment.by organization.

**Table 4:** Results of the models of the mediation effects of physical environment and health culture on self-rated health and mental health improvement

Potential mediating variables	Model	Self-rated health improvement					Model	Mental health improvement				
		*R* ^2^	Adjusted *R*^2^	*R* ^2^ change	*F* change	*P*		*R* ^2^	Adjusted *R*^2^	*R* ^2^ change	*F* change	*P*
Physical environment	Model A	0.029	0.018	0.029	2.594	*0.008*	Model E	0.081	0.070	0.081	7.584	*< 0.001*
	Model B	0.031	0.018	0.002	1.611	0.205	Model F	0.082	0.070	0.000	0.310	0.578
Health culture	Model C	0.020	0.008	0.020	1.738	0.086	Model G	0.075	0.064	0.075	6.874	*< 0.001*
	Model D	0.048	0.036	0.028	20.580	*< 0.001*	Model H	0.090	0.078	0.014	10.603	*0.001*

All the models were adjusted for gender, age, education level, and marital status

Statistical significance was set at 0.05

**Table 5:** Parameter estimates of models of the mediating effects of physical environment and health culture on self-rated health improvement

Potential mediating variables		Variables	Self-rated health improvement		
			Unstandardized coefficients	Standardized coefficients	*P*
Physical environment	Model A	Constant	2.001		0.154
		Male	−0.053	−0.025	0.531
		Age	0.003	0.026	0.578
		Married	0.048	0.014	0.724
		High school/technical secondary school	0.160	0.047	0.403
		Junior college	0.154	0.063	0.366
		Bachelor or higher degree	0.181	0.085	0.272
		Intervention implementation	0.345	0.108	*0.005*
		Physical environment	−0.062	−0.110	*0.004*
Health culture	Model D	Constant	13.470		*< 0.001*
		Male	−0.026	−0.013	0.755
		Age	0.003	0.025	0.603
		Married	−0.011	−0.003	0.935
		High school/technical secondary school	0.184	0.054	0.345
		Junior college	0.216	0.088	0.212
		Bachelor or higher degree	0.185	0.087	0.274
		Intervention implementation	−3.249	−1.014	*< 0.001*
		Health culture	−3.570	−2.204	*< 0.001*
		Health culture × intervention implementation	0.860	2.698	*< 0.001*

Statistical significance was set at 0.05

**Table 6:** Parameter estimates of models of the mediating effects of physical environment and health culture on mental health improvement

Potential mediating variables		Variables	Mental health improvement		
			Unstandardized coefficients	Standardized coefficients	*P*
Physical environment	Model E	Constant	4.784		0.546
		Male	−0.471	−0.039	0.317
		Age	0.040	0.065	0.158
		Married	−1.209	−0.062	0.116
		High school/technical secondary school	2.086	0.108	*0.046*
		Junior college	2.326	0.164	*0.012*
		Bachelor or higher degree	3.041	0.251	*0.001*
		Intervention implementation	3.681	0.200	*< 0.001*
		Physical environment	−0.367	−0.114	*0.003*
Health culture	Model H	Constant	45.123		*0.022*
		Male	−0.300	−0.025	0.529
		Age	0.034	0.056	0.232
		Married	−1.494	−0.078	*0.050*
		High school/technical secondary school	2.482	0.127	*0.020*
		Junior college	2.762	0.194	*0.004*
		Bachelor or higher degree	3.116	0.256	*0.001*
		Intervention implementation	−11.328	−0.616	*0.015*
		Health culture	−14.343	−1.549	*0.002*
		Health culture × intervention implementation	3.544	1.947	*0.001*

Statistical significance was set at 0.05

Table 5 shows that intervention implementation was significantly positively correlated with SRH improvement. However, a higher physical environment score was significantly negatively associated with SRH improvement. The interaction between health culture and intervention implementation was particularly noteworthy. When the interaction between health culture and intervention implementation was included, the interaction was statistically significant with SRH improvement. Thus, the parameter estimates of model D could not directly indicate the effects of variables; these effects needed to be calculated. Holding the intervention implementation score still, with a 1-unit increase in health culture, there was a change in the SRH improvement value of 0.860 × intervention − 3.570. Therefore, we expect a positive association between health culture and the SPH improvement when the intervention implementation score exceeded 4.151.

With respect to mental health improvement, results of model E show that higher education level and intervention implementation were significantly positively correlated with mental health improvement. However, a higher physical environment score was significantly negatively associated with mental health improvement. Results of model H show that higher education level was significantly positively correlated with mental health improvement, while marriage was negatively associated with mental health improvement. Moreover, the interaction between health culture and intervention implementation was also significant in model H. Holding the intervention implementation score still, with a 1-unit increase in health culture, there was a change in the mental health improvement value of 3.544 × intervention − 14.343. Therefore, we expect a positive association between health culture and the mental health improvement when the intervention implementation score exceeded 4.047. In our study, the average intervention implementation score was 4.229 out of 5; meanwhile, 6 of the 10 workplaces had an intervention implementation score higher than 4.151.

## Discussion

After a two-year comprehensive intervention, the means of SRH and mental health increased significantly by 0.302 and 2.698, respectively. Higher intervention effectiveness was observed in our study than in some previous studies [21, 22]. The difference could be due to differences in the study design [23]. Other possible explanations include management support and highly targeted interventions. For example, each workplace established a health promotion leadership team to help ensure effective health promotion. Moreover, the comprehensive interventions targeted priority problems determined by the need assessments of each workplace. Compared with other intervention studies, our intervention duration was longer [22, 24]. The goal of most workplace health promotion programs has been to improve health behaviors in China, and few studies have targeted self-rated health and mental health, making it difficult to compare their results to ours [25, 26].

Regarding the correlates of intervention effectiveness, there were no demographic variables correlated with SRH improvement. However, results suggest that education level and marital status were correlated with mental health improvement. Participants with high education level were more likely to benefit from intervention programs focusing on mental health. The possible reason might be that the participants with high education level may be more likely to accept and understand the interventions related to mental health [27]. For marriage, the same intervention on mental health improvement for married employees was not as effective as unmarried employees. It might be attributable to the more impact of work on unmarried employees than married employees.

Besides the demographic correlates, the association between intervention implementation and intervention effectiveness was also examined. In an effort to evaluate intervention implementation quantitatively, we used the Chinese Workplace Health Scorecard. The scores may reflect the quality of the intervention, including its overall health promotion and specialized health promotion. The results showed that intervention implementation was significantly positively correlated with intervention effectiveness. This was consistent with the findings of other studies [28–30].

It is worth noting that our study focused on the role of physical environment and health culture on intervention effectiveness. We examined whether the physical environment could mediate the relationship between intervention implementation and self-rated health and mental health improvement. Results showed that physical environment did not mediate the relationship between intervention implementation and intervention effectiveness. Furthermore, physical environment was significantly negatively correlated with self-rated health and mental health improvements, which was inconsistent with some previous studies [31]. The lack of study effect might be attributed to study methods or the way physical environment was measured. First, the results of the baseline evaluation show that the better the physical environment, the better the employees’ mental health. Thus, the intervention might have been less effective. Second, we did not measure the changes in physical environment scores since the physical environment was included as a mediating variable in the study. Third, the direct observation method to assess the physical environment was the objective measurement of dose delivered; while collecting information from participants about the physical environment would be capturing dose received which was not assessed. By not collecting dose received measurements, some information was left unknown that may have potentially explained why the physical environment was significantly negatively correlated with self-reported health and mental health.

Another finding from our study could be more valuable: Health culture may mediate the relationship between intervention implementation and SRH and mental health improvement. Although the importance of health culture has been mentioned in some previous studies [11, 32], few quantitative studies have been conducted to examine its impact. One qualitative study found that organizational culture had a primary influence on the implementation of health-promoting initiatives; when the existing culture was positive towards healthy behaviors, the implementation of health-promoting initiatives was easily supported [11]. Our findings supported this view with data: As long as the intervention was above a certain score, the better the health culture, the better the intervention effectiveness in terms of both SRH and mental health. According to the Workplace Health Culture Scale [19], there was one item assessing the support from workplace by “Resources are provided to support health promotion”. Thus, one of the reasons of significant interactions between health culture and intervention implementation might be leadership support for employee health which was confirmed by several studies [33, 34]. On the other side, leadership support mainly referred to policy support, human resources and funding in previous studies [33, 35], while, the assessment of health culture mainly focuses on the individual’s and direct leadership’s health behaviors and their influence [20]. As defined by the Workplace Health Culture Scale, there are five dimensions of workplace health culture: individual health culture, adverse health behaviors of direct leadership, adverse health effects of direct leadership, beneficial health effects of direct leadership, and overall health culture [19]. The scale can be divided into three levels: the individual, the direct leadership, and the workplace. The impact of the direct leadership on the health culture is investigated with detailed items such as “My direct leader likes smoking” and “My direct leader persuades me to smoke”. It is clear that a good health culture requires not only leadership support for policies and resources, but also leading by example and encouraging employees to adopt a healthy lifestyle. Therefore, health culture might mediate the relationship between workplace health promotion and intervention effectiveness by creating healthy social norms.

Compared to the physical environment, health culture was more important for workplace health promotion in our study. This finding is consistent with some previous qualitative studies [10, 11]. There are two possible reasons for the difference in the importance of these factors. First, the impact of workplace health culture on the implementation of health promotion was comprehensive (including policies and programs). In this context, health culture referred to the attitudes, practices, and social norms (smoking, drinking, physical activity) regarding health within the workplace [11]. However, the impact of the physical environment on employees’ health was supportive—for example, if no one was willing to exercise, increasing exercise facilities would not be helpful. Second, workplace health culture had a broader impact on the health behaviors of employees, while the role of the physical environment was limited to the workplace. For example, the smoke-free environment in the workplace was only useful for limiting the employees’ smoking behaviors in the workplace, and had little effect on smoking behavior outside the workplace. However, a smoke-free culture in an organization might change employees’ smoking behavior more completely. Therefore, under relatively high-quality interventions with scores higher than 4.047 or 4.151, better health culture can lead to better SRH and mental health improvements.

Considering the good intervention effectiveness in our study, there are several practical implications for SRH and mental health improvement. Firstly, it should be a priority to set a series of health policies in workplace such as smoke-free policy and work-break exercises policy. Secondly, it is important to establish an effective working mechanism in workplace health promotion such as assembling a healthy workplace team. A healthy workplace team is not only the embodiment of the health promotion, but also the key force of the practical health promotion. Thirdly, the need assessments are necessary for the development of interventions. Finally, given that health culture can mediate the relationship between workplace health promotion and intervention effectiveness, it is essential to create a good health culture by setting health policies, establishing an effective working mechanism, and seeking leadership support.

There are some limitations to our study. First, for practical reasons, a control group was not established, which limits the significance of the intervention evaluation. Second, the sample was only from civil servants, so the ability to extrapolate the results to the larger workforce is limited. Third, because some variables including physical environment and workplace health culture were not measured consistently at each of the three time periods, we failed to determine for example if there were any floor or ceiling effects in play or to test for directionality on the role of health culture. Fourth, it would have been a more complete process evaluation had both dose delivered and dose received been collected. It would have allowed the authors to see whether or not worksites with higher physical environment scores (dose delivered) correlated with the participants’ engagement (dose received). Last, a two-level hierarchical linear regression analysis should have been employed as respondents were clustered within workplaces. However, the results of testing showed that our data appeared not applicable to multi-level analysis. References to the results of testing are also given with the Additional file 4.

## Conclusions

Health culture mediated the relationship between workplace health promotion and intervention effectiveness, but physical environment did not. Under relatively high-quality interventions, a better health culture may lead to more positive improvements in SRH and mental health. Our results also suggest several practical implications for SRH and mental health improvement such as setting health policies, establishing a working mechanism, conducting need assessments, and creating a good health culture. Future studies will need to examine the physical environment as a moderating effect rather than mediating effect. Moreover, more researches should be expanded to different types of workplaces.

## Additional files

Additional file 1: Study Protocol. (PDF 652 kb)

Additional file 2: Direct Observation Scoring Table for workplace. (PDF 99 kb)

Additional file 3: **Table S1.** Demographic characteristics of participants by organization. **Table S2.** Differences in gender, marital status, and education among the participants across the 10 organizations. **Table S3.** Differences in age and length of service among the participants across the 10 organizations. **Table S4.** Descriptive statistics and t test results of SRH and mental health by organization. **Table S5.** Physical environment by organization. **Table S6.** Descriptive results for intervention implementation measured by Chinese Workplace Health Scorecard. **Table S7.** Descriptive results for workplace health culture measured by Workplace Health Culture Scale. **Table S8.** Descriptive results for workplace physical environment measured by Direct Observation Scoring Table. (PDF 298 kb)

Additional file 4: Supplementary materials for data testing. (PDF 34 kb)

## Abbreviations

CDC: Centers for Disease Control and Prevention
SRH: Self-rated health
WHO: World Health Organization
