# Supplementary material for: The roles of health culture and physical environment in workplace health promotion: a two-year prospective intervention study in China
Source: BMC Public Health. 2018 Apr 5;18:457. doi: 10.1186/s12889-018-5361-5 (PMC5887264; doi:10.1186/s12889-018-5361-5)
Supplement: Supplementary file 1 — Study Protocol. (PDF 652 kb) [file 12889_2018_5361_MOESM1_ESM.pdf]

Study Protocol

1. Protocol name: The study on the influencing factors and interventive effectiveness of worksite health promotion
2. Study Design:

2.1 Hypothesis:

Hypothesis A: Whether the environmental (physical environment and culture) and organizational factors (leadership support and employee participation) can mediate the relationship between worksite health promotion and effect.

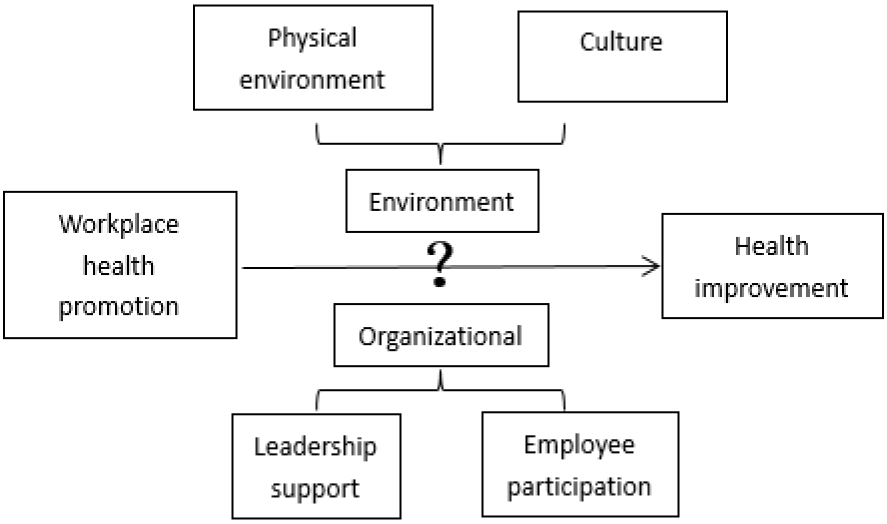

Hypothesis A

Hypothesis B: Explore the intermediate variables between health promotion interventions and changes in health status.

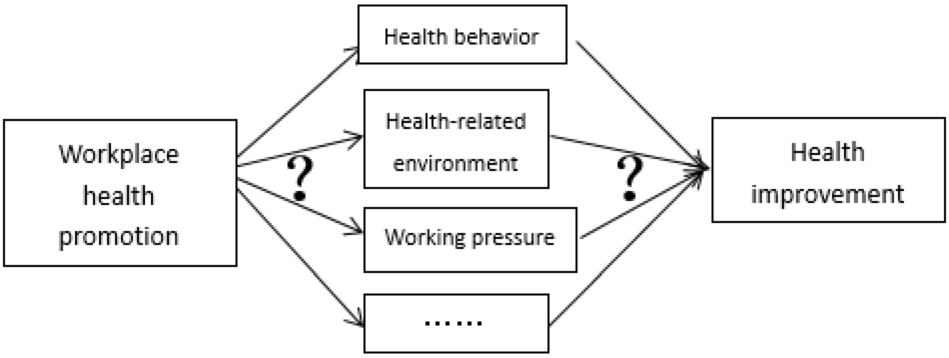

Hypothesis B

2.2 Objectives

Overall objective: To examine whether the environmental and organizational factors can mediate the relationship between worksite health promotion and effect and explore the intermediate variables between health promotion interventions and changes in health status; explore an integrated model for worksite health promotion based on the case

study.

Specific objectives: Assess the effects of interventions; analyze the factors that affect the health promotion effects of the workplace; compare the different characteristics of health promotion effects and conduct a simple case study.

2.3 Study design

A prospective self-controlled design was employed and 10 government agencies were selected in the present study. In August 2012, the needs assessment was carried out for ten workplaces, and the baseline data were collected. Then, according to the results of need assessment, the main health problems and the priorities of the intervention for each workplace were determined. After that, targeted intervention advices were provided. In September 2013, a process assessment was conducted for ten workplaces, focusing on the implementation of the interventions and recommending further improvements based on the current situation. In September 2014, the final evaluation was carried out for ten workplaces, and the terminal data were collected. In order to explore a health promotion integrated model, the health-related indicators of the same participants were compared with each other before and after the intervention, and the effect of the intervention was evaluated.

2.4 Theoretical framework

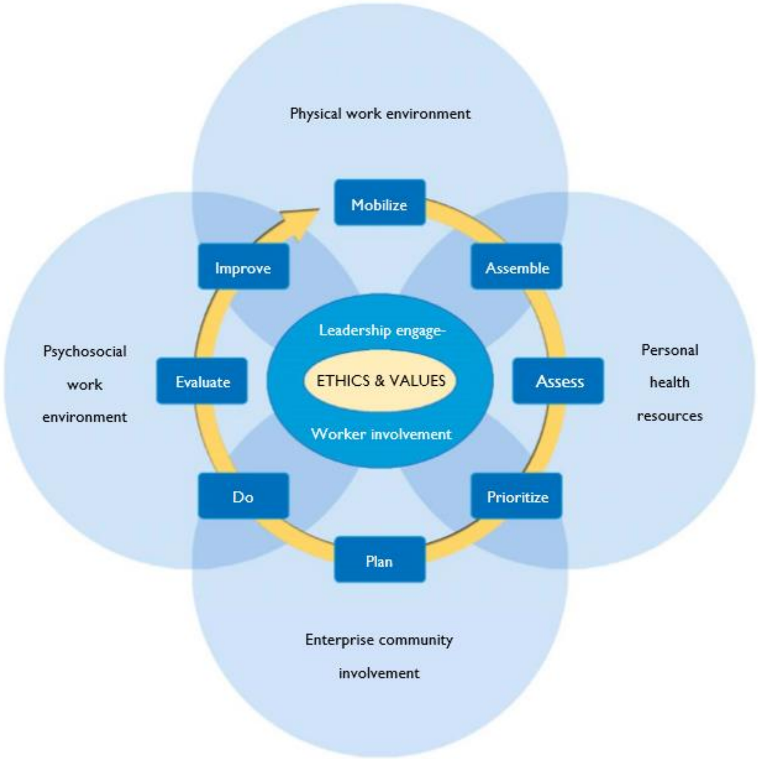

3. Innovation

(1) It is the first study to introduce the observation method to evaluate the material environment for the effect of intervention in workplace health promotion.

(2) It is the first study to assess the health culture of the workplace and to evaluate its impact on the effectiveness of health promotion interventions in the workplace.

(3) Innovative application of quantitative methods to assess the impact of leadership support and employee participation on health effects in workplace health

promotion.

(4) Innovatively through the experimental design of interventions to explore possible intermediate variables between health promotion interventions and changes in health status.

(5) It is the first intervention study to carry out systematic process assessment in order to accurately assess the impact of intervention factors in workplace health promotion.

Methods

1. Participants

This study selects 10 government departments in Shanghai, including employees who sign any form of contract with the workplace (long-term contract workers, temporary workers, labor dispatch workers, etc.), not including travel during the investigation, sick leave, or refused to participate in the investigation. Among 10 selected workplaces, eight were the town government in the suburbs of Shanghai, and two were the neighborhood offices in Shanghai's central city.

2. Methods

2.1 Sample size calculation

2.2 Data collection

This study was a prospective self-control intervention study. Based on signing informed consents, all the staff of the government agencies with similar size (about 100 people) were selected as the basic sampling group, and the qualified staff members were included. On the basis of extensive mobilization, the staff need to complete the questionnaires themselves. The informed consent is the cover of the questionnaire to inform the rights and obligations of the respondents, and promised to keep the contents of the questionnaire.

According to the overall design of the study, data collection mainly includes baseline assessment, process assessment, and effectiveness assessment.

2.2.1 Baseline assessment

Needs assessment will be conducted to collect the baseline information, and determine the main health problems of each workplace from July to September 2012. The main survey method is as follows.

2.2.1.1 Questionnaire investigation

(1) Content of questionnaire

According to literature and expert's advice, questionnaire content of needs assessment has been determined. The questionnaire is divided into five parts: basic demographic characteristics, health status, behavioral risk factors, job-related influencing factors and health promotion need.

① Demographic characteristics: Gender, age, marital status, education, length of service, and type of employment.

② Health status: SRH, chronic disease, and mental health.

③ Health behavior: Smoking, passive smoking, physical activity, healthy diet, health knowledge, and sleep.

④ Work-related factor: Social capital, stress, and social support.

⑤ health promotion need: Health promotion activity, time, organization, and information channel.

(2) Survey method

The staff responsible for workplace health promotion need to receive training. The unified

design, unified printed self-administered questionnaires will be distributed to employees. If the employee does not understand the subject matter in the questionnaire, he may consult with the investigator immediately, but the investigator may not answer any content that involves the answer to the question or mislead or direct the employee to fill in the answer.

2.2.1.2 Qualitative interviews

Each person responsible for workplace health promotion need to receive an interview. The main contents include the basic situation of employees, the main problems of health, the health factors, the basic situation of tobacco control and nutrition diet, the health promotion activities carried out, and the health promotion activities in future.

2.2.2 Process assessment

We will carry out process assessment for ten workplaces, focus on assessing the specific implementation of intervention measures, leadership support, and employee participation from September to December 2013. After that, we will make suggestions for further improvement according to the current situation.

2.2.2.1 Questionnaire investigation

(1) Content of questionnaire

According to WHO Health Workplace Model, combined with the actual implementation of the workplace health intervention measures, the contents of the process assessment questionnaire will be determined. The questionnaire is divided into four parts: basic demographic characteristics, health status, health culture, health promotion activities.

① Demographic characteristics: Gender and age.

② Health status: SRH.

③ Health culture: Workplace health culture and organization health.

④ Participation in health promotion activities: Health promotion programs, individual participation, participation of colleagues, participation of direct leaders, and participation of key leaders.

(2) Survey method

Since the focus of the process assessment is to assess the overall health promotion interventions of the workplace, all respondents in the workplace at the time of the assessment are not same with the staff at the baseline survey. The unified design, unified printed self-administered questionnaires will be distributed to employees. If the employee does not understand the subject matter in the questionnaire, he may consult with the investigator immediately, but the investigator may not answer any content that involves the answer to the question or mislead or direct the employee to fill in the answer.

2.2.2.2 Direct observation

(1) Content:

① Overall situation: Overall health situation, health promotion column, and use of health promotion column.

② Physical activity environment: Space for exercise, fitness equipment, and usability of staircase.

③ Tobacco control environment: Smoking point set, smoking equipment in office, and tobacco control signs posted.

④ Nutritional diet environment: Canteen status, diet provision, and publicity of food knowledge.

(2) Observation methods and scoring

① Observation methods: Based on field observation and inquiry, the two observers rate independently.

② Scoring: A total of four dimensions, each dimension has three items. The total score of each item is 5 points, while the overall score is 60 points.

2.2.2.3 Qualitative interviews

(1) Leadership support scale

By interviewing the leader of each workplace, we will understand the support of the leadership for workplace health promotion. Assessment content is divided into four dimensions as follows.

① Business alignment with health promotion objectives
② Awareness of link between health and worker productivity
③ Worksite support for health promotion
④ Leadership support for health promotion

(2) Workplace health promotion scorecard

By interviewing the person responsible for health promotion, the actual situation of workplace health promotion in the past year is assessed. Assessment content is divided into six dimensions as follows.

① Management support
② Tobacco control
③ Nutrition diet
④ Physical activity
⑤ Bodyweight control
⑥ Stress management

2.2.3 Effectiveness assessment

In order to explore a health promotion integrated model, the health-related indicators of the same participants will be compared with each other before and after the intervention, and the effect of the intervention will be evaluated. Effectiveness assessment will be conducted from September to November 2014.

2.2.3.1 Questionnaire investigation

(1) Content of questionnaire

In order to compare with the baseline survey results, the effectiveness assessment questionnaire as a whole is similar with the baseline questionnaire. The questionnaire is divided into five parts: basic demographic characteristics, health status, behavioral risk factors, job-related influencing factors and participation in health promotion activities.

(2) Survey method

Since the effectiveness assessment will focus on assessing the effectiveness of the intervention, the survey will be conducted primarily for employees who participated in the baseline assessment and add some new respondents. The survey method is the same as above.

2.2.3.2 Direct observation

The method of observation and scoring is the same as the process assessment.

2.2.3.3 Qualitative interviews

(1) Leadership support scale: the same as the process assessment.

(2) Workplace health promotion scorecard: the same as the process assessment.

(3) Stakeholder interviews

① Management: Management mainly refers to the leaders involved in decision making. Leader of each workplace is invited to accept a personal in-depth interviews.

② Health promotion stuff: Health promotion stuff mainly refers to the persons involved in the implementation of health promotion plan. Health promotion stuff of each workplace is invited to accept a personal in-depth interviews.

③ Employee representative: Among the employees, 6-8 employee representatives selected by convenience sampling join in the focus group interview.

2.3 Indicators and definition

2.3.1 Demographic characteristics

(1) Gender: Dichotomous variable, divided into male and female.

(2) Age: Continuous variable, the four age categories are <30, 30–39, 40–49, and ⩾50 years.

(3) Marital status: Dichotomous variable, divided into married and unmarried/divorced/widowed.

(4) Education: Ordinal categorical variable, The four education categories are junior high school, high school/technical secondary school, junior college, and bachelor/master's/doctorate degree.

(5) Length of service: Continuous variable, the four categories are <5, 5–14, 15–24, and ⩾25 years.

(6) Type of employment: Categorical variable, divided into civil servants, career preparation, temporary workers, other types of preparation.

2.3.2 Health status

(1) SRH: Respondents will be asked to rate their own general health on a five-point scale ranging from perfect to poor. Self-rated health score can be included as a continuous variable into the analysis, but also SRH can be divided into two categories of variables, "perfect", "very good", and "good" into "good SRH" , while "fair" and "poor" into "fair and poor SRH".

(2) Chronic diseases: Ten common diseases is listed, including hypertension, diabetes, fatty liver, hyperlipidemia, cervical spondylosis, coronary heart disease, bronchitis, kidney disease, stroke, cancer. In the follow-up analysis, the prevalence of chronic diseases is divided into two categories.

(3) Mental health: Mental health was measured by the Chinese version of WHO-Five Well-being Index. We used 5 questions to investigate the status of respondents over the past 2 weeks. For instance, how often have you felt cheerful and in good spirits? We used 5 questions to investigate the status of respondents over the past 2 weeks. Respondents answered each question on a 6-point scale ranging from never (0) to all the time (5). According to the total scores <13 points or ⩾ 13 points, the respondents were divided into "poor mental health" or "good mental health", respectively.

2.3.3 Health behavior

(1) Smoking: Smoking status is divided into "current smokers ", "ex-smokers", "non-smokers". Smokers are classified as respondents who had smoked more than 100 cigarettes. Ex-smokers are classified as respondents who had smoked more than 100 cigarettes and are under smoking cessation.

(2) Passive smoking: Passive smokers are respondents who had been exposed to others' smoke for more than 15 minutes in the last week

(3) Alcohol intake was dichotomized with 1 representing yes, and 0 representing no.

(4) Physical activity: The IPAQ short form asked about three specific types of physical activity (PA) including walking, moderate-intensity activities such as dancing, cycling, and performing tai chi, and vigorous-intensity activities such as swimming and playing basketball. The following values were used to analyze IPAQ data: walking = 3.3 metabolism equivalents (METs; moderate PA = 4.0 METs, and vigorous PA = 8.0 METs). Calculating of the total score for the short form required summation of the duration (in minutes) and frequency (days) of walking, moderate-intensity activities, and vigorous-intensity activities. Three levels of physical activity were proposed to classify the populations: low, moderate, and high. The two criteria for classification as "high" were (a) vigorous-intensity activity on at least 3 days achieving a minimum total physical activity of 1500 MET-minutes per week, or (b) 7 or more days of any combination of walking, moderate-intensity, or vigorous-intensity activities achieving a minimum total physical activity of 3000 MET-minutes per week. The pattern of

activity classified as “moderate” was either of the following criteria: (a) 3 or more days of vigorous-intensity activity of at least 20 minutes per day, (b) 5 or more days of moderate-intensity activity and/or walking for at least 30 minutes per day, or (c) 5 or more days of any combination of walking, moderate-intensity, or vigorous intensity activities achieving a minimum total physical activity of 600 MET-minutes per week. Individuals who did not meet the criteria for “moderate” or “high” were considered to have a “low” level of physical activity.

(5) Nutrition diet: Ask the survey respondents about the daily diet of the individual and the taste of meals provided by workplace, the options are very salty, a little salty, moderate, a little light, and very light. In the follow-up analysis, the individual taste variable from five categories into three categories, that is salty, moderate, and light. Regarding to the oil of meals provided by workplace, the options are very oil, a little oil, moderate, a little light, and very light. In the follow-up analysis, the individual oil variable from five categories into three categories, that is oil, moderate, and light.

(6) Health knowledge: Health knowledge is composed of four questions including daily recommended intake of salt, oil, vegetables, and fruit. Calculate the scores of health knowledge based on whether the answer is correct or not.

(7) Sleep: Divided into "insomnia" and "normal sleep" two categories. Divided into "insomnia" and "normal sleep" two categories. Ask the survey about three questions about sleep time, sleep depth, morning wake. If you sleep too long (more than 30 minutes), the depth of sleep is not enough (more than 3 times a week often wake up), morning awake too early (more than 3 times a week to wake up too early and can not sleep again), as long as one of the three answers is positive, you can determine the insomnia.

2.3.4 Work-related factors

(1) Social capital: The social capital scale measures both the cognitive and structural components of social capital using eight items. The internal consistency of the scale was good (Cronbach’s alpha = 0.88). A summary score of the ratings of all social capital items is constructed, in which a high score indicates high social capital.

(2) Work pressure and social support: Questions regarding job demand, job control, and support at work are based on the Karasek’s Job Content Instrument. Five factors are used to measure the psychological demand of the job: working very carefully, working very fast, using a lot of information, freedom from conflicting demands, and requests to do an excessive amount of work. Decision latitude is assessed using four questions about the employee’s ability to use and develop skills (by asking if the job involves learning new things, non-repetitive work, creativity, and a high skill level) and exert authority (by assessing freedom to decide how to perform work, and the ability to make one’s own decisions). Professional support is assessed by four questions regarding support from colleagues, supervisors, family, and workplace. Based on the results of job demand and control, we can calculate the occupational stress score according to the following formula. Occupational stress score = job requirements equalization / job autonomy. If the score of occupational stress > 1.0 is occupational stress positive.

2.3.5 Implementation of intervention

(1) Workplace health promotion scorecard

Combined with China's national conditions, the scorecard is developed based on the workplace health promotion scorecard designed by US Centers for Disease Control and Prevention. The scorecard is divided into two parts, the overall implementation of health promotion and the implementation of special work.

① Overall implementation: Overall implementation is measured by 11 items ranged from 1 to 5 points. Higher score indicates better implementation of health promotion.

② Implementation of special work: Implementation of special work includes 5 dimensions. Tobacco control is measured by 10 items. Higher score indicates better implementation of tobacco control. Healthy diet is measured by 12 items. Higher score indicates better implementation of Healthy diet. Physical activity is measured by 10 items. Higher score indicates better implementation of physical activity. Bodyweight control is measured by 10 items. Higher score indicates better implementation of bodyweight control. Stress management is measured by 10 items. Higher score indicates better implementation of stress management.

(2) Human resource and funding

As a potential influencing factor of effectiveness, human resource and funding is assessed by interviewing leader and health professionals of each workplace.

2.3.6 Influencing factor of intervention effectiveness

(1) Leadership support:

① Leadership support scale: A total of 13 items, each item ranges from1 to 5 points, the total score of all items is the leader support.

② Organizational support: A total of 5 items are based on workplace health promotion scorecard including leadership commitment, attitude of health promotion committee, plan , target, and funding guarantee. Each item ranges from1 to 5 points.

③ Leadership participation in health promotion programs: it is divided into direct leaders and key leaders. Leadership participation is divided into non-participation, participation, occasional participation, regular participation, and always attendance, assigned to 1-5 points. Index of leadership participation= direct leadership participation + key leadership participation.

(2) Employee participation: Employee participation is divided into no participation, participation, occasional participation, frequent participation, and always participation, respectively, assigned to 1-5 points. Index of employee participation= individual participation+ colleagues' participation.

(3) Workplace physical environment: The direct observation scoring table is divided into four parts, including the overall situation, physical activity environment, tobacco control environment, and nutritional diet environment.

(4) Workplace health culture and organizational health: The scale included 20 items, which were divided into five dimensions: individual health culture, adverse health behaviors of direct leadership, adverse health effects of direct leadership, beneficial health effects of direct leadership, and overall health culture. Each item was rated on a scale from 1 to 5, with a higher score indicating better workplace health culture. The scores of the five dimensions were averaged to obtain the total average score, which was included in the analysis as a continuous variable. The effects of workplace health culture were derived from the intervention effectiveness evaluation. Organizational health scale consists of 9 items.

2.4 Interventions and effectiveness assessment

2.4.1 Determination of health problem

(1) Need assessment

The priority problems were determined based on prevalence, correlation with other risk factors, possibility of making a difference, and consistency between management's ideas and the views of employees.

Table 1. Priority of health problem

Risk factor	Prevalence	Correlation	Possibility of change	Consis tency	Total score	Priority
Smoking/Passive smoking	4	2	4	1	11	2

Physical activity	2	3	5	2	12	1
Stress/Insomnia	3	1	4	2	10	3
Health knowledge	1	1	5	2	9	4

(2) Risk assessment

According to the results of physical examination, seven kinds of common chronic diseases were assessed for group risk, including lung cancer, liver cancer, esophageal cancer, gastric cancer, diabetes, coronary heart disease and stroke. Risk score = baseline incidence rate × relative risk, risk of onset - population total incidence rate × combined risk score, therefore, the combination of risk score is a rough estimate of the risk of individual or group. Based on the calculation method of the risk assessment and the distribution of measurable risk factors for each disease, the following three types of risk scores are calculated.

① The existing risk score refers to the current employee in the presence of a risk factor combination of risk scores.

② The post-change risk score refers to the combined risk score after the current employee change all the risk factors that can be changed, including the smoking cessation of the employee, the passive smoking, and the active adjustment of the mentally unhealthy employee.

③ The changeable risk score = the existing risk score – post-change risk score. It refers to the current employee change all the risk factors can be changed after the original reduction of the combined risk score. And then sorted by reducing the changeable risk score, and ultimately prioritize the intervention.

According to the classification of risk factors, calculate the existing risk factors for each risk factor, post-change the risk score and the changeable risk score, by comparing the risk factors can reduce the size of the risk score, the overall intervention priority is determined. The specific results are shown in Table 2.

Table 2. Priority of risk factors

Changeable	Risk factors	Existing risk score		Total existing risk score	Post-change risk score		Total post-change risk score	Changeable risk score		Total changeable risk score	intervention priority
		Male	Female		Male	Female		Male	Female		
Changeable	Passive smoking	120.32	94.966	215.286	58.201	50.858	109.059	62.119	44.108	106.227	1
	Drinking	169.157	152.877	322.034	103.945	125.09	229.035	65.212	27.787	92.999	2
	Smoking	212.614	163.807	376.421	120.203	163.807	284.01	92.411	0	92.411	3
	Hyperlipidemia	101.16	65.81	166.97	44.012	43.536	87.548	57.148	22.274	79.422	4
	Mental health	75.096	72.014	147.11	52.377	52.23	104.607	22.719	19.784	42.503	5
	Overweight	102.983	75.212	178.195	71.713	66.213	137.926	31.27	8.999	40.269	6
	Physical activity	75.897	71.055	146.952	63.66	56.65	120.31	12.237	14.405	26.642	7
	High salt diet	22.68	20.58	43.26	21.7	19.6	41.3	0.98	0.980	1.96	8
Unchangeable	Hypertension	77.521	76.059	153.58	77.521	76.059	153.58	0	0	0	
	Diabetes	95.533	87.819	183.352	95.533	87.819	183.352	0	0	0	

2.4.2 Development of intervention

Based on literature, a standard intervention plan is provided for each workplace including physical activity, tobacco control, healthy diet, and working stress as follows.

Table 3. Intervention strategy for physical activity

Objective	Strategy	Level	Manager	Process assessment
1. Awareness of recommendation of physical activity	➢ lectures on physical activity related knowledge and skills<br>➢ Post posters about knowledge	Workplace	Health staff	➢ Frequency and effectiveness (employee participation)<br>➢ Poster coverage
2. Active transportation Promotion	➢ Encourage the staff close to workplace go to work by foot or bicycle<br>➢ Parking fees need to pay if the staff is close to workplace	Workplace	Leaderships	➢ Proportion of changing transportation
3. Promotion for leisure-time physical activity	➢ Add health paths and equipment<br>➢ Improve working system for physical activity<br>➢ Work-break exercises<br>➢ Praise for active employees<br>➢ Provide pedometer and tips for exercise places	Workplace	Leaderships / health staff	➢ Physical condition and use of health paths<br>➢ Implementation of new working system<br>➢ Participation in work-break exercises<br>➢ Praise<br>➢ Number of pedometers
	➢ Tips for reducing sedentary behavior<br>➢ Interest group of exercise<br>➢ Daily physical activity<br>➢ Encourage to raise habit of regular exercise by email and message	Group	Employee	➢ Communication<br>➢ Organization of self-management<br>➢ Frequency of daily activity<br>➢ Frequency of email and message
4. Promotion for stair use	➢ Improve environment of stair and stair use<br>➢ Decision point	Workplace	Health staff	➢ Usability of stairs<br>➢ Number of tips
5. Ergonomic related measures	➢ Add ergonomic equipment<br>➢ Spread knowledge of ergonomics	Workplace	Leaderships	➢ Number of ergonomic equipment

Table 4. Intervention strategy for tobacco control

Objective	Strategy	Level	Manager	Process assessment
1. Smoke-free policy	➢ Set leading organizations for tobacco control and establish smoke-free system;<br>➢ Arrange supervision and implementation;<br>➢ Awareness of smoke-free policy;<br>➢ Key leader announced to start the creation of smoke-free workplace	Workplace	Leaderships	➢ Set leading organizations ；<br>➢ Responsibility of key leader；<br>➢ Whether the system meets the principle of 100% no smoke<br>➢ Implementation of reward and punishment policy<br>➢ Records of supervision<br>➢ Ways to propagate Smoke-free policy<br>➢ Awareness of employees who understand smoke-free policy
2. Supportive environment	➢ Remove smoking paraphernalia;<br>➢ Poster no smoking tips;<br>➢ Play ads for smoke-free;<br>➢ No tobacco sales and no ads for tobacco;	Workplace	Leaderships / health staff	➢ Number of smoking paraphernalia；<br>➢ Number of no smoking tips；<br>➢ Frequency of playing ads for smoke-free
3. Improve awareness of smoking and passive smoking	➢ lectures on smoking;<br>➢ Various ways of ads for smoke-free<br>➢ Training for all the employee from different level	Workplace	Health staff	➢ Content, method, and coverage；<br>➢ Satisfaction
4. Improve smokers' willingness to quit smoking and behavior	➢ Provide training for smokers<br>➢ Encourage smoker to get help from 12320<br>➢ Encourage smoker to go to Quit smoking clinic<br>➢ Encourage smoker's family to help them quit smoking	Workplace	Leaderships / health staff	➢ Participation in quit smoking training；<br>➢ Satisfaction for training<br>➢ Implementation of new policy<br>➢ Number of employee who get help from 12320<br>➢ Proportion of calling 12320<br>➢ Feedback from smoker's family

	➢ Encourage smoker to quit smoking in pair<br>➢ Sign a smoking cessation contract with a smoker	Group	Leaderships of group	➢ Number of pairs<br>➢ Number of contracts

Table 5. Intervention strategy for healthy diet

Objective	Strategy	Level	Manager	Process assessment
1. Awareness of knowledge about healthy diet	➢ Provide knowledge about healthy diet；<br>➢ Posters about healthy diet；<br>➢ Provide training for employees；	Workplace	Health staff	➢ Coverage and frequency of information；<br>➢ Coverage of poster；<br>➢ Frequency of lecture；
2. Creation of healthy canteen	➢ Post a leaflet for unhealthy diet；<br>➢ Carry out "cooking competition"	Workplace	Leaderships / health staff	➢ Coverage of leaflet；<br>➢ Participation in "cooking competition"；
	➢ Training for food procurement；<br>➢ Training for cook including health knowledge and skills；<br>➢ Keep dining environment clean and poster the recommended total calorie intake；<br>➢ Provide healthy food with discount；<br>➢ Provide special food for employees with chronic disease If necessary；	Canteen	Leaders and management of canteen	➢ Coverage of canteen；<br>➢ Frequency of training；<br>➢ Situation of health menu；<br>➢ Record of salt and oil；<br>➢ Improvement of environment;

	➢ Record the use of salt and oil monthly；<br>➢ Collect advises from employees ；			➢ Awareness of knowledge about healthy diet among cooks；<br>➢ Improvement of health diet<br>➢ Health outcome such as bodyweight and fat；
3. Encourage employee to raise healthy habit for diet	➢ Encourage employee to eat in the cafeteria ；<br>➢ Provide service for recommended take - away food；<br>➢ Weighing scale；	Workplace	Leaderships	➢ Development of recommended take - away food；<br>➢ Use of weighing scale；
4. Self-management for high-risk	➢ Train the leaders of group;<br>➢ Teach employee to develop own health diet<br>➢ Provide healthy food	Workplace	Health staff	➢ Training；<br>➢ High-risk participation；<br>➢ Frequency of activities

Table 5. Intervention strategy for working pressure

Objective	Strategy	Level	Manager	Process assessment
1. Improve awareness of knowledge	➢ Improve communication between colleagues<br>➢ Provide information timely<br>➢ Regularly hold a health meeting	Individual	Leaderships / health staff	➢ Frequency of lecture<br>➢ Employee participation<br>➢ Ways and coverage of information
2. improve skill	➢ Skill training about communication and exercise<br>➢ Provide necessary consulting service	Individual	Leaderships / health staff	➢ Content and frequency of training<br>➢ Frequency of consulting service
3. improve communication, social network, and social support	➢ Establish a variety of interest groups<br>➢ Organize activities based on family<br>➢ Help each other to cope with family problem	Workplace	Leaderships / health staff	➢ Creation of interest groups<br>➢ Situation of activities

	➢ Encourage employee to share working experience	Group	Leaders of group	➢ Communication
	➢ Regularly hold summary meetings			➢ Situation of summary meetings
	➢ Division and cooperation			
4. Improve health culture and physical environment	➢ Create health climate	Workplace	Health staff	➢ Creation and organizing of interest groups
	➢ Regularly organize health promotion activities			➢ Policy development
	➢ Encourage team-work	Workplace	Leaderships	➢ Funding support
	➢ Develop related policy for group activity			➢ Environmental support
	➢ Provide funding for interest group			
	➢ Provide supportive environment			

Based on the standard intervention plan, actual situation and need assessment, targeted intervention plan is determined by each workplace. After the implementation of the intervention for a year, according to the process assessment, the intervention program will be developed. The flowchart is shown as follows.

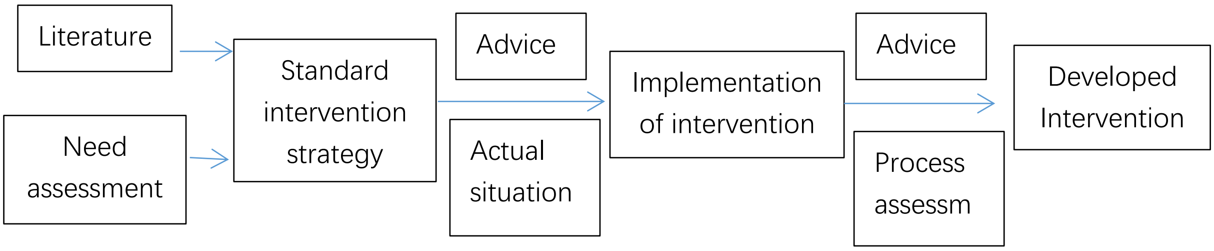

Figure. Flowchart of intervention development

2.4.3 Implementation of intervention

(1) At the start of the program, we recruited the top management of each government agency that committed to the program. The agencies agreed to invest essential human and financial resources into health promotion.Each workplace assembled a "healthy workplace team", and had several members as well as financial support dedicated to health promotion.

(2) Focusing on the key problems, each workplace made targeted efforts to promote employees' health. Some common interventions were as follows:

① For tobacco control, each workplace set up regulations such as "Smoking is banned indoors", "No smoking in the office", "Leaders take the lead in smoking cessation", and "Posting no-smoking signs".

② For physical activity, space was expanded, more exercise facilities were provided, some interest groups were organized with financial support, and employees were encouraged to walk rather than take the elevator by posters hung at the decision points.

③ For nutrition and diet, salt and oil dosage were recorded every working day; healthy eating habits were introduced through means such as lectures, leaflets, posters, and videos; food was colored with red, yellow, and green for recommending intake.

④ For stress management, social activities were organized with financial support, and lectures about mental health were held.

2.4.4 Effectiveness assessment

Intervention effectiveness assessment is divided into two levels.

(1) Individual level: Compare the baseline and final data of the same participants.

① Health status: Improvement of SRH and mental health;

② Health behavior: Improvement of smoking, passive smoking, physical activity, nutrition and diet, health knowledge, and sleep.

③ Work-related factors: improvement of social capital, work pressure and social support.

(2) Workplace level: Compare the baseline and final data of the same workplace.

① Health status: Improvement of mean of SRH and mental health;

② Health behavior: Improvement of prevalence of smoking, passive smoking, physical activity, nutrition and diet, health knowledge, and sleep.

③ Work-related factors: Improvement of mean of social capital, work pressure and social support.

2.5 technology roadmap

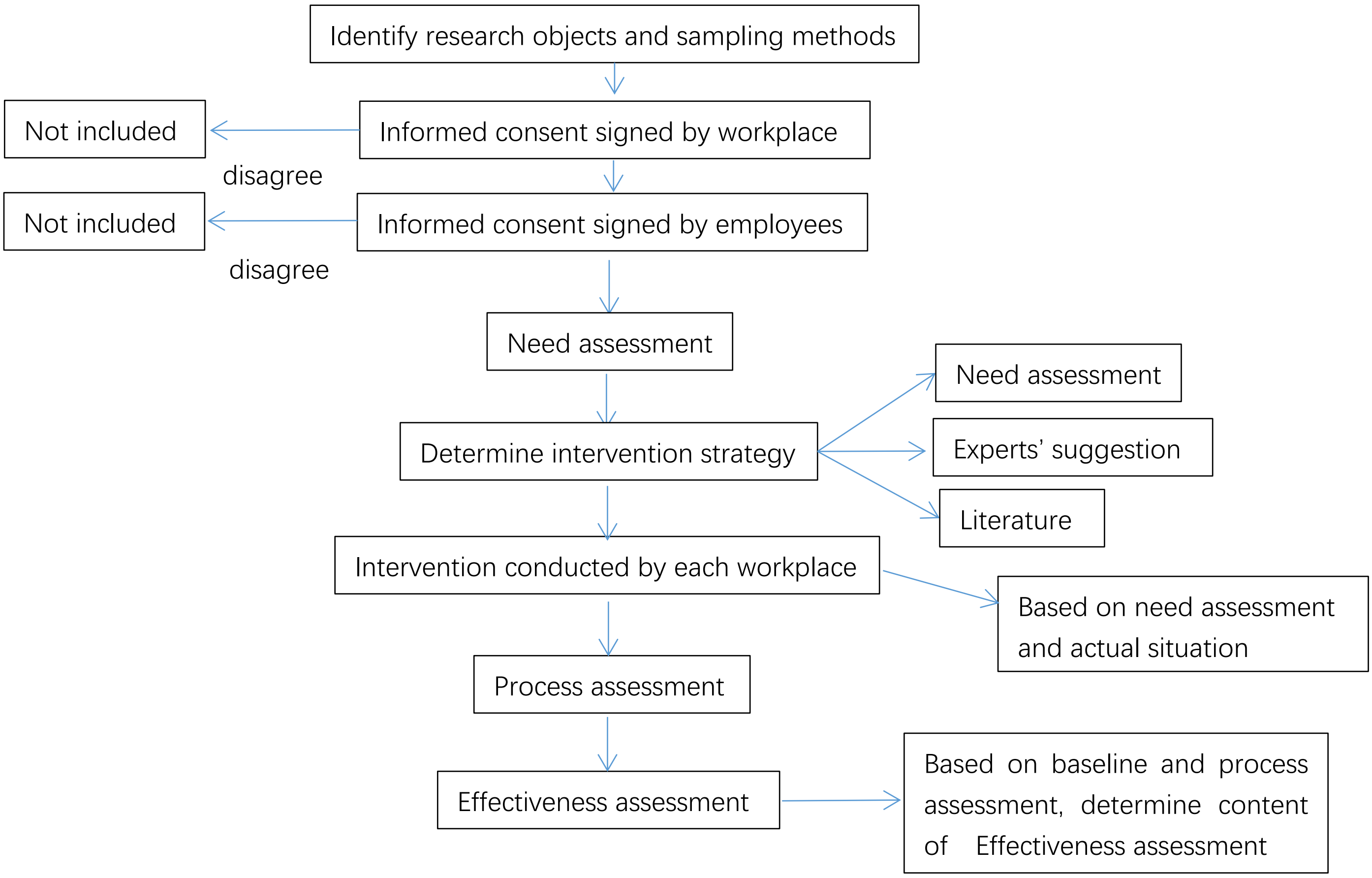

2.6 Quality Control

2.6.1 Questionnaire investigation

The questionnaires are self-filled by the respondents. Trained investigators are responsible for on-site quality control, including answering questions and asking respondents to complete the missing items on the questionnaires. No response rate should be less than 20%.

2.6.2 Qualitative interviews

Interviews are conducted by experienced interviewers. Interviews for leaders, health professionals and employee representatives are conducted at different time and locations to prevent the interaction between them. All interviews are recorded on the site, followed by the project staff independently on the recording verbatim sentence translated into manuscript.

2.6.3 Direct observation

According to the Direct Observation Scoring Table, each trained observer rates the health environment of the workplace independently through field observation.

2.7 Analyses

2.7.1 Quantitative data

Descriptive analyses, t-tests, and multiple regression were conducted for the quantitative data using Epidata 3.1, Excel 2010, and Statistical Package for Social Sciences 20.0. To evaluate the effectiveness of the intervention, a paired t-test was used first; then, repetitive measurement and analysis of variance were performed. Multiple regression analysis was conducted to determine whether the health culture and physical environment could mediate the relationship between intervention implementation and intervention effectiveness.

2.7.2 Qualitative data

The qualitative data is collected by the form of free organization text. Content and subject analysis method is employed using QSR Nvivo7 software. The researchers read all the documents alone, analysis of words and text blocks, coding, classification, respectively. Finally, the interpretation of the subject is determined, while the conclusion can be obtained.
