# Supplementary material for: The roles of health culture and physical environment in workplace health promotion: a two-year prospective intervention study in China
Source: BMC Public Health. 2018 Apr 5;18:457. doi: 10.1186/s12889-018-5361-5 (PMC5887264; doi:10.1186/s12889-018-5361-5)
Supplement: Supplementary file 2 — Direct Observation Scoring Table for workplace. (PDF 99 kb) [file 12889_2018_5361_MOESM2_ESM.pdf]

Table. Direct Observation Scoring Table for workplace

Workplace：________________________

Judge：______________ **Time**：___________

**1. Overall environment**	**Full score**	**Ratings**
The overall situation of sanitation	5	
Allocation of health bulletin board	5	
Using and updating for health bulletin board	5	
**2. Physical activity environment**	**Full score**	**Ratings**
There is enough space for exercises	5	
There are various fitness equipment	5	
Good availability of the stairwell	5	
**1. Tobacco control environment**	**Full score**	
The settings of smoking area are reasonable	5	
No smoking ashtrays in the office	5	
There are obvious signs for tobacco control	5	
**4. Nutritional/dietary environment**	**Full score**	**Ratings**
Sanitary condition of dining hall is good	5	
Balanced diets, vegetables, and fruits are provided	5	
Knowledge about healthy diet is well promoted	5	
**Total score**	**60**	
