# Supplementary material for: The roles of health culture and physical environment in workplace health promotion: a two-year prospective intervention study in China
Source: BMC Public Health. 2018 Apr 5;18:457. doi: 10.1186/s12889-018-5361-5 (PMC5887264; doi:10.1186/s12889-018-5361-5)
Supplement: Supplementary file 3 — Table S1. Demographic characteristics of participants by organization. Table S2. Differences in gender, marital status, and education among the participants across the 10 organizations. Table S3. Differences in age and length of service among the participants across the 10 organizations. Table S4. Descriptive statistics and t test results of SRH and mental health by organization. Table S5. Physical environment by organization. Table S6. Descriptive results for intervention implementation measured by Chinese Workplace Health Scorecard. Table S7. Descriptive results for workplace health culture measured by Workplace Health Culture Scale. Table S8. Descriptive results for workplace physical environment measured by Direct Observation Scoring Table. (PDF 298 kb) [file 12889_2018_5361_MOESM3_ESM.pdf]

Additional results

Table 1. Demographic characteristics of participants by organization

Workplace	Number of participants	Variable	Mean	Standard deviation	Min	5th percentile	95th percentile	Max
Workplace 1	89	Gender	1.40	0.49	1	1	2	2
		Age	42.46	10.16	24	26	58	63
		Marital status	1.0	0.29	1	1	2	2
		Education	3.33	0.81	1	2	4	4
Workplace 2	64	Gender	1.67	0.47	1	1	2	2
		Age	33.17	7.28	25	26	49	56
		Marital status	1.11	0.31	1	1	2	2
		Education	3.83	0.42	2	3	4	4
Workplace 3	77	Gender	1.44	0.50	1	1	2	2
		Age	41	9.23	23	27	56	58
		Marital status	1.04	0.19	1	1	1	2
		Education	3.16	1.01	1	1	4	4
Workplace 4	90	Gender	1.43	0.50	1	1	2	2
		Age	41.22	8.40	25	29	54	59
		Marital status	1.02	0.15	1	1	1	2
		Education	3.16	1.17	1	1	4	4
Workplace 5	83	Gender	1.46	0.50	1	1	2	2
		Age	41.46	10.42	22	26	58	62
		Marital status	1.05	0.21	1	1	1	2
		Education	2.99	0.97	1	1	4	4
Workplace 6	73	Gender	1.49	0.50	1	1	2	2
		Age	37.37	10.39	23	24	54	59
		Marital status	1.16	0.37	1	1	2	2
		Education	3.18	0.99	1	1	4	4
Workplace 7	56	Gender	1.52	0.50	1	1	2	2
		Age	38.96	9.92	24	25	56	57
		Marital status	1.11	0.31	1	1	2	2
		Education	3.45	0.97	1	1	4	4
Workplace 8	63	Gender	1.57	0.50	1	1	2	2

		Age	41.27	9.25	24	27	54	57
		Marital status	1.16	0.37	1	1	2	2
		Education	3.73	0.54	2	3	4	4
Workplace 9	61	Gender	1.34	0.48	1	1	2	2
		Age	38.75	8.67	22	25	54	57
		Marital status	1.13	0.34	1	1	2	2
		Education	3.20	1.12	1	1	4	4
Workplace 10	63	Gender	1.49	0.50	1	1	2	2
		Age	39.33	10.13	23	24	54	57
		Marital status	1.25	0.44	1	1	2	2
		Education	3.54	0.62	2	2	4	4

For gender, 1 represents male, and 2 represents female. For marital status, 1 represents married, and 2 represents unmarried/divorced/widowed. For education, 1, 2, 3 and 4 represent junior high school, high school/technical secondary school, junior college, and bachelor or higher degree, respectively.

Table 2. Differences in gender, marital status, and education among the participants across the 10 organizations

Organization	Gender		Marital status		Education			
	Male n (%)	Female n (%)	Married n (%)	Unmarried/divorced/widowed n (%)	Junior high school n (%)	High school/technical secondary school n (%)	Junior college n (%)	Bachelor or higher degree n (%)
Workplace 1	53(59.6)	36(40.4)	81(91.0)	8(9.0)	4(4.5)	7(7.9)	34(38.2)	44(49.4)
Workplace 2	21(32.8)	43(67.2)	57(89.1)	7(10.9)	0	1(1.6)	9(14.1)	54(84.4)
Workplace 3	43(55.8)	34(44.2)	74(96.1)	3(3.9)	8(10.4)	10(13.0)	21(27.3)	38(49.4)
Workplace 4	51(56.7)	39(43.3)	88(97.8)	2(2.2)	17(18.9)	4(4.4)	17(18.9)	52(57.8)
Workplace 5	45(54.2)	38(43.3)	79(95.2)	4(4.8)	6(7.2)	21(25.3)	24(28.9)	32(38.6)
Workplace 6	37(50.7)	36(49.3)	61(83.6)	12(16.4)	5(6.8)	15(20.5)	15(20.5)	38(52.1)
Workplace 7	27(48.2)	29(51.8)	50(89.3)	6(10.7)	5(8.9)	4(7.1)	8(14.3)	39(69.6)
Workplace 8	27(42.9)	36(57.1)	53(84.1)	10(15.9)	0	3(4.8)	11(17.5)	49(77.8)
Workplace 9	40(65.6)	21(34.4)	53(86.9)	8(13.1)	9(14.8)	6(9.8)	10(16.4)	36(59.0)
Workplace 10	32(50.8)	31(49.2)	47(74.6)	16(25.4)	0	4(6.3)	21(33.3)	38(60.3)
Total	376(52.3)	343(47.7)	643(89.4)	76(10.6)	54(7.5)	75(10.4)	170(23.6)	420(58.4)
χ^2 (p)	19.884 (0.019)		33.015 (<0.001)		1145(<0.001)			

Table 3. Differences in age and length of service among the participants across the 10 organizations

Organization	Age (years)						Length of service (years)					
	N	Mean (SD)	<30<br>n (%)	30–39<br>n (%)	40–49<br>n (%)	≥50<br>n (%)	N	Mean (SD)	<5<br>n (%)	5–14<br>n (%)	15–24<br>n (%)	≥25<br>n (%)
Workplace 1	89	42.46(10.16)	8(9.0)	30(33.7)	22(24.7)	29(32.6)	89	11.72(8.75)	21(23.6)	40(44.9)	17(19.1)	11(12.4)
Workplace 2	64	33.17(7.28)	21(32.8)	33(51.6)	7(10.9)	3(4.7)	64	5.72(5.23)	32(50.0)	26(40.6)	5(7.8)	1(1.6)
Workplace 3	77	41.00(9.23)	7(9.1)	27(35.1)	25(32.5)	18(23.4)	77	11.43(7.91)	12(15.6)	42(54.5)	16(20.8)	7(9.1)
Workplace 4	90	41.22(8.40)	7(7.8)	30(33.3)	36(40.0)	17(18.9)	90	10.99(7.76)	18(20.0)	49(54.4)	15(16.7)	8(8.9)
Workplace 5	83	41.46(10.42)	11(13.3)	27(32.5)	25(30.1)	20(24.1)	83	10.41(7.22)	16(19.3)	51(61.4)	10(12.0)	6(7.2)
Workplace 6	73	37.37(10.39)	21(28.8)	22(30.1)	18(24.7)	12(16.4)	73	7.23(6.40)	32(43.8)	30(41.1)	9(12.3)	2(2.7)
Workplace 7	56	38.96(9.92)	16(28.6)	12(21.4)	17(30.4)	11(19.6)	56	10.48(7.98)	13(23.2)	27(48.2)	12(21.4)	4(7.1)
Workplace 8	63	41.27(9.25)	10(15.9)	13(20.6)	26(41.3)	14(22.2)	63	11.56(9.70)	20(31.7)	26(41.3)	8(12.7)	9(14.3)
Workplace 9	61	38.75(8.67)	10(16.4)	20(32.8)	25(41.0)	6(9.8)	61	9.07(7.37)	21(34.4)	25(41.0)	14(23.0)	1(1.6)
Workplace 10	63	39.33(10.13)	18(28.6)	8(12.7)	25(39.7)	12(19.0)	63	10.52(7.98)	16(25.4)	30(47.6)	14(22.2)	3(4.8)
Total	719	39.72(9.74)	129(17.9)	222(30.9)	226(31.4)	142(19.7)	719	10.02(7.90)	201(28.0)	346(48.1)	120(16.7)	52(7.2)
F (p) / χ^2 (p)			5.768 (<0.001) / 92.225 (<0.001)						4.633 (<0.001) / 62.337 (<0.001)			

Table 4. Descriptive statistics and t test results of SRH and mental health by organization

Organization	Self-rated health						Mental health					
	N	Mean (SD)					N	Mean (SD)				
		SRH (baseline)	SRH (final)	SRH improvement	t	p		Mental health (baseline)	Mental health (final)	Mental health improvement	t	p
Workplace 1	89	2.85 (0.79)	3.10 (0.92)	0.25 (0.95)	2.468	0.016	88	14.22 (4.87)	18.64 (4.83)	4.42 (6.68)	6.207	<0.001
Workplace 2	64	3.03 (0.84)	3.23 (0.81)	0.20 (0.86)	1.894	0.063	62	14.92 (5.31)	16.45 (4.18)	1.53 (5.01)	2.410	<0.019
Workplace 3	77	3.00 (0.80)	3.03 (0.86)	0.03 (0.92)	0.248	0.804	76	15.91 (4.50)	17.21 (3.78)	1.30 (5.74)	1.977	0.052
Workplace 4	85	2.94 (0.88)	3.24 (1.08)	0.29 (1.15)	2.352	0.021	88	15.02 (4.83)	17.47 (5.81)	2.44 (5.87)	3.903	<0.001
Workplace 5	83	2.89 (0.91)	3.40 (0.92)	0.51 (1.20)	3.831	<0.001	79	14.61 (4.87)	17.71 (4.77)	3.10 (5.50)	5.008	<0.001
Workplace 6	73	3.07 (0.84)	3.68 (0.91)	0.62 (1.14)	4.628	<0.001	69	15.22 (5.22)	18.77 (5.38)	3.55 (6.17)	4.778	<0.001
Workplace 7	56	3.14 (0.80)	3.14 (0.88)	0.00 (0.99)	0.000	1.000	56	14.66 (4.95)	16.05 (4.16)	-1.39 (5.07)	-2.058	0.044
Workplace 8	62	2.76 (0.76)	2.89 (0.79)	0.13 (0.76)	1.342	0.185	60	14.13 (5.42)	16.67 (4.61)	2.53 (5.00)	3.927	<0.001
Workplace 9	61	2.89 (0.78)	2.92 (0.86)	0.03 (0.93)	0.275	0.784	61	14.95 (5.07)	16.26 (4.84)	1.31 (4.79)	2.138	0.037
Workplace 10	62	3.05 (0.86)	3.95 (1.14)	0.90 (1.08)	6.573	<0.001	57	12.74 (5.54)	20.39 (3.32)	7.65 (5.52)	10.460	<0.001
Total	712	2.96 (0.83)	3.26 (0.97)	0.30 (1.05)	7.712	<0.001	696	14.79 (5.02)	17.49 (4.92)	2.70 (5.98)	11.91	<0.001

Table 5. Physical environment by organization

Organization	Overall environment	Physical activity environment	Tobacco control environment	Nutritional/dietary environment	Total scores
Workplace 1	13.56	13.44	14.06	14.88	55.94
Workplace 2	13.75	14.63	14.00	14.75	57.00
Workplace 3	14.25	13.94	14.75	14.75	57.69
Workplace 4	14.00	14.88	14.88	15.00	58.75
Workplace 5	14.13	12.13	13.50	13.88	53.63
Workplace 6	13.81	14.94	13.69	13.75	56.19
Workplace 7	14.25	13.50	14.00	14.50	56.25
Workplace 8	14.38	13.00	14.75	14.00	56.13
Workplace 9	13.88	11.88	14.00	13.88	53.63
Workplace 10	12.88	11.81	13.75	14.13	52.56
Average scores	13.89	13.42	14.14	14.35	55.78
t (p)	100.349 (<0.001)	35.315 (<0.001)	92.092 (<0.001)	95.833 (<0.001)	90.604 (<0.001)

Table 6. Descriptive results for intervention implementation measured by Chinese Workplace Health Scorecard

Workplace	Overall health promotion Mean		Specialized health promotion Mean		Total score of intervention implementation Mean	
	Collection point		Collection point		Collection point	
	12 months	24 months	12 months	24 months	12 months	24 months
Workplace 1	4.250	4.583	4.800	4.826	4.525	4.705
Workplace 2	3.583	4.667	3.730	4.734	3.657	4.701
Workplace 3	4.083	4.167	4.228	3.624	4.156	3.896
Workplace 4	4.083	4.500	3.948	4.694	4.016	4.597
Workplace 5	3.750	4.333	3.594	4.090	3.672	4.212
Workplace 6	4.667	4.833	4.554	4.602	4.611	4.718
Workplace 7	4.000	3.417	4.302	3.280	4.151	3.349
Workplace 8	2.833	4.083	3.494	4.612	3.164	4.348
Workplace 9	4.583	4.250	4.674	4.454	4.629	4.352
Workplace 10	4.417	4.833	4.284	4.720	4.351	4.777
Average	4.025	4.367	4.161	4.364	4.093	4.365

Table 7. Descriptive results for workplace health culture measured by Workplace Health Culture Scale

Workplace	Individual health culture Mean(SD)	Adverse health behaviors of direct leadership Mean(SD)	Adverse health effects of direct leadership Mean(SD)	Beneficial health effects of direct leadership Mean(SD)	Overall health culture Mean(SD)	Total average score of workplace health culture Mean(SD)
	Collection point 24 months	Collection point 24 months	Collection point 24 months	Collection point 24 months	Collection point 24 months	Collection point 24 months
Workplace 1	4.62 (0.55)	3.99 (1.35)	4.06 (1.22)	4.52 (0.62)	4.56 (0.58)	4.35 (0.63)
Workplace 2	4.41 (0.64)	4.01 (1.24)	3.89 (1.15)	4.00 (0.75)	4.09 (0.74)	4.09 (0.54)
Workplace 3	4.46 (0.52)	4.06 (1.13)	4.21 (1.11)	4.28 (0.63)	4.37 (0.58)	4.27 (0.54)
Workplace 4	4.41 (0.71)	3.89 (1.11)	3.75 (1.14)	4.26 (0.80)	4.26 (0.75)	4.11 (0.69)
Workplace 5	4.09 (0.86)	2.87 (1.30)	3.04 (1.28)	3.91 (0.84)	3.89 (0.74)	3.56 (0.60)
Workplace 6	4.50 (0.54)	3.99 (1.37)	3.92 (1.31)	4.34 (0.57)	4.44 (0.56)	4.23 (0.62)
Workplace 7	4.44 (0.52)	3.83 (1.09)	3.86 (1.07)	4.12 (0.66)	4.22 (0.61)	4.09 (0.55)
Workplace 8	4.54 (0.58)	3.89 (1.42)	3.98 (1.34)	4.45 (0.66)	4.66 (0.52)	4.30 (0.56)
Workplace 9	4.41 (0.62)	3.69 (1.41)	4.08 (1.02)	4.07 (0.71)	4.40 (0.71)	4.13 (0.59)
Workplace 10	4.80 (0.38)	4.21 (1.46)	4.31 (1.23)	4.72 (0.56)	4.70 (0.62)	4.55 (0.66)
Average	4.46 (0.63)	3.83 (1.33)	3.89 (1.24)	4.27 (0.73)	4.35 (0.69)	4.16 (0.65)

Table 8. Descriptive results for workplace physical environment measured by Direct Observation Scoring Table

Workplace	Overall environment Mean		Physical activity environment Mean		Tobacco control environment Mean		Nutritional/dietary environment Mean		Total score of workplace physical environment Mean	
	Collection point		Collection point		Collection point		Collection point		Collection point	
	12 months	24 months	12 months	24 months	12 months	24 months	12 months	24 months	12 months	24 months
Workplace 1	13.75	13.375	14.25	12.625	14.50	13.625	15.00	14.75	57.5	54.375
Workplace 2	13.25	14.25	15.00	14.25	13.75	14.25	14.75	14.75	56.75	57.5
Workplace 3	13.75	14.75	13.25	14.625	14.50	15.00	14.75	14.75	56.25	59.125
Workplace 4	14.50	13.50	15.00	14.75	14.75	15.00	15.00	15.00	59.25	58.25
Workplace 5	14.00	14.25	12.00	12.25	12.50	14.50	14.00	13.75	52.5	54.75
Workplace 6	14.00	13.625	15.00	14.875	13.50	13.875	14.50	13.00	57	55.375
Workplace 7	14.00	14.50	13.00	14.00	13.25	14.75	14.50	14.50	54.75	57.75
Workplace 8	14.00	14.75	12.50	13.50	15.00	14.50	13.50	14.50	55	57.25
Workplace 9	14.00	13.75	12.50	11.25	14.00	14.00	13.50	14.25	54	53.25
Workplace 10	12.50	13.25	12.00	11.625	13.50	14.00	14.00	14.25	52	53.125
Average	13.81	13.97	13.52	13.41	13.95	14.36	14.40	14.36	55.68	56.1
