# Supplementary material for: The roles of health culture and physical environment in workplace health promotion: a two-year prospective intervention study in China
Source: BMC Public Health. 2018 Apr 5;18:457. doi: 10.1186/s12889-018-5361-5 (PMC5887264; doi:10.1186/s12889-018-5361-5)
Supplement: Supplementary file 4 — Supplementary materials for data testing. (PDF 34 kb) [file 12889_2018_5361_MOESM4_ESM.pdf]

Supplementary materials for data testing

① For self-rated health improvement, we found that ICC (intraclass correlation coefficient)= 0.07003/(0.07003+1.0327)=0.0635. Among all the 10 workplaces, there were only two significant random effect.

Parameter Estimation of Covariance (SRH improvement as a dependent variable)

Covariance parameter	Variable	Parameter Estimation	Standard error
Intercept	Workplace	0.07003	0.04053
Residual		1.0327	0.05513

Fixed effect(SRH as a dependent variable)

Effect	Parameter Estimation	Standard error	Degrees of freedom	t	P
Intercept	0.2971	0.0921	9	3.23	0.0104

Random effect(SRH as a dependent variable)

Effect	Variable	Parameter Estimation	Std Err Pred	t	P
Intercept	Workplace 1	-0.2351	0.1411	-1.67	0.0961
Intercept	Workplace 2	0.4897	0.1378	3.55	**0.0004**
Intercept	Workplace 3	-0.00251	0.1285	-0.02	0.9844
Intercept	Workplace 4	0.1774	0.1292	1.37	0.1699
Intercept	Workplace 5	0.2657	0.1328	2	**0.0458**
Intercept	Workplace 6	-0.07634	0.1368	-0.56	0.577
Intercept	Workplace 7	-0.04278	0.1273	-0.34	0.7369
Intercept	Workplace 8	-0.2128	0.1383	-1.54	0.1244
Intercept	Workplace 9	-0.2275	0.1313	-1.73	0.0835
Intercept	Workplace 10	-0.1357	0.1378	-0.98	0.325

② For mental health improvement, we found that ICC(intraclass correlation coefficient)= 4.9877/(4.9877+31.7975)=0.13559. Among all the 10 workplaces, there were only two significant random effects.

Parameter Estimation of Covariance (mental health improvement as a dependent variable)

Covariance parameter	Variable	Parameter Estimation	Standard error
Intercept	Workplace	4.9877	2.6036
Residual		31.7975	1.7172

Fixed effect(SRH as a dependent variable)

Effect	Parameter Estimation	Standard error	Degrees of freedom	t	P
Intercept	2.6492	0.7387	9	3.59	0.0059

Random effect(SRH as a dependent variable)

Effect	Variable	Parameter Estimation	Std Err Pred	t	P
Intercept	Workplace 1	-3.6289	0.9745	-3.72	0.0002
Intercept	Workplace 2	4.497	0.9711	4.63	<.0001
Intercept	Workplace 3	-0.1921	0.9007	-0.21	0.8312
Intercept	Workplace 4	0.4183	0.9163	0.46	0.6482
Intercept	Workplace 5	0.8253	0.9376	0.88	0.3791
Intercept	Workplace 6	-1.0128	0.9559	-1.06	0.2897
Intercept	Workplace 7	1.6516	0.9007	1.83	0.0671
Intercept	Workplace 8	-1.2112	0.9587	-1.26	0.2069
Intercept	Workplace 9	-1.2424	0.9222	-1.35	0.1784
Intercept	Workplace 10	-0.1047	0.9617	-0.11	0.9133

③ In summary, the results of testing showed that our data appeared not applicable and necessary to multi-level analysis. First, the two ICC were small. Second, the difference between the workplace level was very small and almost not statistical significant. Third, the demographic characteristics of workers from each organization seem to be similar. Therefore, we believe that multiple linear regressions might be suitable.
